# The Importance of Periodic Dental Control in the Oral Health Status of Elderly Patients

**DOI:** 10.3390/clinpract13020050

**Published:** 2023-04-18

**Authors:** Michael Janto, Raluca Iurcov, Cristian Marius Daina, Alina Cristiana Venter, Corina Lacramioara Suteu, Monica Sabau, Dana Badau, Lucia Georgeta Daina

**Affiliations:** 1 Faculty of Medicine and Pharmacy, Doctoral School, University of Oradea, 1 December Sq., 410081 Oradea, Romania; 2 Dentistry Department, Faculty of Medicine, University of Oradea, 410073 Oradea, Romania; 3 Psycho-Neurosciences and Recovery Department, Faculty of Medicine and Pharmacy, University of Oradea, 1 December Sq., 410081 Oradea, Romania; 4 Department of Morphological Sciences, Faculty of Medicine and Pharmacy, University of Oradea, 1 December Sq., 410081 Oradea, Romania; 5 Petru Maior Faculty of Sciences and Letters, George Emil Palade University of Medicine, Pharmacy, Sciences and Technology, 540142 Targu Mures, Romania; 6 Interdisciplinary Doctoral School, Transylvania University, 500068 Brasov, Romania

**Keywords:** epidemiological profile, associated chronic diseases, dental hygiene, accessibility of dental care services, quality of life, dental health and diagnosis, endodontic diagnosis

## Abstract

The aim of the present study was to evaluate the status of the elderly patient with oral pathology, comparing two groups of elderly patients, from the point of view of presentation for periodic dental control (regular and irregular). In carrying out the study, the following parameters were analyzed: the epidemiological profile of the group of patients; associated chronic diseases; dental hygiene; the accessibility of, and attendance at, dental health services; quality of life; and dental clinical diagnosis. Of the 120 elderly patients included in the study, only 25% present regularly for dental checkups. Increasing age leads to a reduction in the share of patients who regularly present themselves for dental checkups, and patients with higher education and those with higher incomes regularly visit the dentist. Associated chronic diseases are found in both groups; patients with regular checkups have a statistically significantly lower drug consumption for dental pain in the last month compared to those who present irregularly for checkups. A better achievement of dental hygiene is observed in patients who regularly see the dentist. It is alarming that we find patients (10% among those who do not visit the dentist regularly) who do not use a toothbrush and toothpaste, and approximately 40% of the patients enrolled in the study do not know which toothpaste they use. Approximately 40% of the interviewed patients indicate a lack of money as the main reason for attending the dentist only when necessary. Difficult access is mentioned by 10% of the group of patients who regularly visit the dentist and who come from rural areas. The reasons for visiting the dentist are different in the two groups: patients who regularly visit the dentist request caries treatment or prosthetic work, or they present with dental bleeding; patients with irregular checkups especially request emergency services such as toothache treatment, assistance with denture problems, and tooth extractions. When undergoing odontal and endodontic examination, 60% of the respondents who go to the dentist as required suffer from periodontal disease, and 50% of them suffer from class I and II edentation. The results of the study demonstrated that elderly patients who regularly attend periodic dental checkups have better self-reported and dentist-confirmed oral health status.

## 1. Introduction

Over time, new values have been added to health so that today we can look at health from three perspectives: as a right, as a consumer good, and as an investment [1,2,3]. The right to health is guaranteed by the WHO (World Health Organization) Constitution [4], the consumer good is generated by the individual perspective as a material aspect, and the investment in health is important from the point of view of the productive capacity of the labor force. How the three components are prioritized by each country’s government causes differences in health provision.

Oral health is a component of general health and according to the resolution adopted in May 2021 by the General Assembly of the World Health Organization (World Health Assembly) it represents a global health priority [5,6]. The factors that influence health are also prominently found in oral health, being grouped into four categories [7,8]: genetic factors (e.g., genetic inheritance, special needs conditions, a family history of periodontal disease); environmental factors (e.g., socioeconomic status—educational level, occupation, income; physical factors; biological factors—microorganisms, toxins; chemical factors—industrial waste, pollution); behavioral factors—lifestyle (e.g., diet, alcohol and tobacco consumption, consumption of sweets, poor oral hygiene); and organization of health services (availability, acceptability, accessibility, quality of medical services, etc.) [9]. Most of the time, we find in an individual several risk factors that interact, for example, poor hygiene associated with an unhealthy diet and alcohol and tobacco consumption contribute to the occurrence of oral cavity diseases [10]. Oral health has a considerable influence on general health; studies conducted in recent years demonstrate the direct correlation between oral infections (with the systemic release of cytokines or microbial products) and various systemic diseases (diabetes, cardiovascular diseases, Alzheimer’s disease) [11].

In the elderly population, chronic exposure to a multitude of risk factors, as well as the physiological aging processes, causes the appearance of dental pathology, with the most common dental diseases in the elderly being dental caries, periodontal diseases, and stomatitis, along with lesions of the oral mucosa and temporomandibular pathology [12]. With advancing age, a series of physiological and pathological changes occur in the oral cavity, so for an appropriate medical treatment of the elderly patient, the dentist must differentiate between the state of physiological aging (senescence) and the state of the disease. An adaptation of health services is thus required, through the development of medical assistance services that ensure a correct approach to the elderly patient (with or without effects on their oral and/or general state of health). In this population category, an important role is played by secondary and tertiary prevention, health education, and the promotion of a sanogenic lifestyle, provided mainly by the dentist. Improving oral hygiene contributes to reducing the development or occurrence of respiratory diseases [13,14,15], and behavior due to a response to environmental factors (predominantly socioeconomic factors) can be changed in a positive sense through appropriate communication and information. It is important for the individual to be aware of, and to recognize, their responsibility for maintaining and improving health [16].

Periodic dental control involves visiting the dentist every six months or twice in the preceding 12 months [17,18]. The purpose of the research is to evaluate the status of the elderly patient with oral pathology, comparing two groups of elderly patients, from the point of view of presentation for periodic dental control (regular and irregular); investigating existing comorbidities; dental hygiene; the accessibility of, and attendance at, dental health services; quality of life; and clinical dental diagnosis.

## 2. Materials and Methods

### 2.1. Study Design

The present study is part of a larger study aimed at the complex evaluation of dental health status in elderly patients in order to identify the particular aspects that lead to the need to optimize dental medical services, formulating a new managerial model of intervention on levels of prophylaxis. The cross-sectional study was carried out in 2021 (January–December), by means of the distribution and completion by 120 elderly people and 7 attending dentists of a questionnaire made up of 37 questions divided into 6 sections: epidemiological profile (6 questions); associated chronic diseases (5 questions); dental hygiene parameters (9 questions); the degree of accessibility of, and attendance at, dental care services (4 questions); quality of life from the perspective of dental health (6 questions); and odontal and endodontic diagnosis (7 questions). The last section was completed by the dentist after the consultation. The questionnaires were applied to patients who went to 7 dental offices (5 offices in the municipality of Oradea and 2 offices in the metropolitan area of Oradea). Prior to completing the questionnaire, each patient was informed, and the consent of the patients was obtained. The study was conducted in accordance with the Declaration of Helsinki. This study was designed respecting ethical considerations, and it was approved by the University of Oradea, in accordance with document no. 426/11.03.2021.

### 2.2. Participations 

After completing the questionnaires and following the collection of the data, the patients were grouped into two groups according to their periodic dental control: regular (30 patients) and irregular (90 patients). The average age of the patients was 69 ± 3 years, with most patients falling into the 65–69 age group (80 patients—66.67%). Some 24 patients (20.0%) fell into the age group 70–74 years, 9 patients (7.5%)—75–79 years, 6 patients (5.0%)—80–84 years, and 1 patient (0.83%)—85–89 years. According to the distribution by gender and environment of origin, 51 patients (42.5%) were male and 69 patients (57.5%) were female, 57 patients (47.5%) came from the urban environment, and 63 patients (52.5%) were from the countryside. In selecting the target group, it was decided to include elderly people (65–89 years old) who participated in completing the self-assessment questionnaire and were examined by dentists. The study was started in 2019 and started from the premise that each dentist should recruit approximately 2 elderly patients per week for 2 years, which would have meant a batch of approximately 1400 patients (2 patients/week × 50 weeks × 7 dentists × 2 years = 1400 patients). The epidemiological situation created by the COVID-19 pandemic, the establishment of the state of emergency (March–May 2020) and the state of alert (May 2020–April 2022) in Romania led to the restriction/decrease of patient access to dental care, and the target group was reduced considerably (see study limits).

### 2.3. Statistical Analysis 

Excel software and Medcalc software were used for statistical analysis; the *p* < 0.05 was considered statistically significant. The results were processed with SPSS 24 using the following statistical parameters: Chi square (Chi^2^) and degrees of freedom (df). Using Chi square, we observed whether there was a statistically significant relationship between variables in relation to the hypothesis of the study.

## 3. Results

Analyzing the patients’ profiles according to the frequency of dental checkups, the results were grouped into six types of sections found in the questionnaire.

### 3.1. Epidemiological Profile

From the six questions in this section, the epidemiological profile, and the comparative analysis according to regular/irregular periodic dental control, no statistically significant differences by gender, environment of origin, and marital status were obtained. With regard to age group, we found significant differences (*p* < 0.001) for the 70–74 year old group, where we found that 50% of all patients regularly visit the dental office, compared to 10% who only go when necessary. Some 10% of patients with irregular periodic checkups and patients with no regular periodic checkups (*p* = 0.07) are in the 75–79 age group. As regards educational level, 50% of patients with regular periodic dental control have higher education vs. 16.67% of those with irregular dental control (*p* = 0.003).

According to the incomes achieved per month, statistically significant differences are found in those with low incomes, where 50% of patients with irregular periodic control have incomes between EUR 100 and EUR 300 vs. 20% of those with regular dental control (*p* = 0.004), and 10% of patients with regular periodical control have incomes below EUR 100 vs. no patient with irregular control (*p* = 0.002) (Table 1). The percentage of patients with monthly incomes greater than EUR 300 is higher in the group that regularly presents itself for periodic dental checkups.

### 3.2. Associated Chronic Diseases

In this section, general health status was assessed by analyzing the presence of chronic diseases in the elderly population, the diagnosis of the main disease, the consumption of medications, and the number of medications administered daily, as well as whether medications for dental pain were consumed in the last month. From the self-assessment obtained following the administration of the questionnaire, 50% of the elderly patients declared that they suffer from various chronic diseases, the most common being cardiovascular diseases (42 patients). Daily drug consumption was found in 72 of the patients (60%), and 45 patients (37.5%) consumed between one and three drugs daily. Statistically significant differences were recorded only in the question related to the use of drugs for dental pain in the last month, where 36.67% of patients who do not regularly visit the dentist answered yes vs. 20% of patients who present themselves regularly for periodic control. Additionally, 80% of patients with regular periodical dental checkups did not consume drugs for dental pain vs. 63.33% of those with irregular dental control (*p* = 0.09) (Table 2).

### 3.3. Dental Hygiene Aspects

With regard to dental hygiene, most patients use toothpaste and a toothbrush: 100% of patients who present for regular periodic checkups and only 80% of patients who present irregularly for periodic checkups (*p* = 0.0082). Only in the group of patients who do not regularly visit the dentist do we find that they only use water/rinse their mouths (10%) (*p* = 0.07). We find patients in both groups who use combined methods in oral hygiene.

The frequency of tooth brushing is reported as daily by100% of patients who present themselves regularly for periodic checkups, compared to 83.33% of those who present themselves irregularly. We find statistically significant differences in the case of brushing twice a day: 40% regular control vs. 10% irregular control (*p* = 0.0002). None of the interviewed patients brush their teeth after every meal; instead, there are statistically significant differences in tooth brushing every evening: 50% of patients with regular dental checkups vs. 20% of those with irregular controls (*p* = 0.001).

The type of toothbrush used is the average one, used by 60% of patients with regular checkups vs. 56.67% patients with irregular controls. Most patients change their toothbrush when needed (45% of patients with regular checkups vs. 50% of patients with irregular checkups), and changing the brush every three months is mentioned by 50% of patients with regular checkups vs. 26.67% of patients with irregular controls. The type of toothpaste frequently used is special toothpaste for the gums (30% vs. 23.33%) and fluoride toothpaste (30% vs. 20%), but most patients state that they do not know which toothpaste they use (40% vs. 43.33%). Bleeding during tooth brushing occurs more frequently in those who present irregularly for dental checkups (46.67%) compared to those who regularly visit the dentist (40%).

Looking at the medical history, 30% of patients with regular checkups declare “often” having problems with their teeth since childhood vs. 36.67% of patients with irregular checkups, and “from time to time” 50% vs. 30%. The main sources of information regarding oral health are the dentist and television shows in both groups of patients (Table 3).

### 3.4. Accessibility to, and Attendance at, the Dentist

Barriers to access to the dentist are more frequently identified by patients who regularly present themselves for regular checkups as a lack of money (40%), a lack of perceived need (30%), and unpleasant experiences from the past (20%), and the group that irregularly attends the dentist listed a lack of perceived need (26.67%), a lack of money (23.33%), fear (23.33%), difficult access (10%), and unpleasant past experiences (10%). Statistically significant differences (*p* < 0.05) between the two groups were obtained for difficult access, where we do not find any respondents from patients who regularly visit the dentist vs. 10% for those who present irregularly to the dentist, as well as for those who mention the lack of money: 40% of patients with regular periodic dental checkups vs. 23.33% of those with irregular dental control (Table 4).

Attending the dentist is motivated by a series of frequently encountered oral health problems being described. These are as follows, comparing regular control with irregular control: toothache (30% vs. 46.67%), caries treatment (20% vs. 10%), gum pain (10% vs. 6.67%), tooth extractions (10% vs. 6.67%), and prosthetic works (20% vs. 6.67%). It is statistically significant that we find attending the dentist for bleeding gums only in the group of patients who present themselves regularly for periodic dental checkups (*p* = 0.002). It is noticeable that the patients in the group who regularly visit the dentist have a much higher attendance for various problems. Likewise, in the selected group of patients, no elderly patient indicates scaling as a reason for contacting the dentist.

Other aspects analyzed were those related to the attendance of the patient at the same dentist, where 90% of patients with regular periodic dental checkups visit the same dentist vs. 83.33% of those with irregular dental control. Periodic control is considered important by 100% of the patients who regularly see the doctor, compared to 80% of those with irregular control (*p* = 0.008). A total of 10% of the patients in the group who present themselves for regular dental checkups do not consider it important (*p* = 0.07).

### 3.5. Quality of Life from a Dental Health Perspective

**Figure 1:**
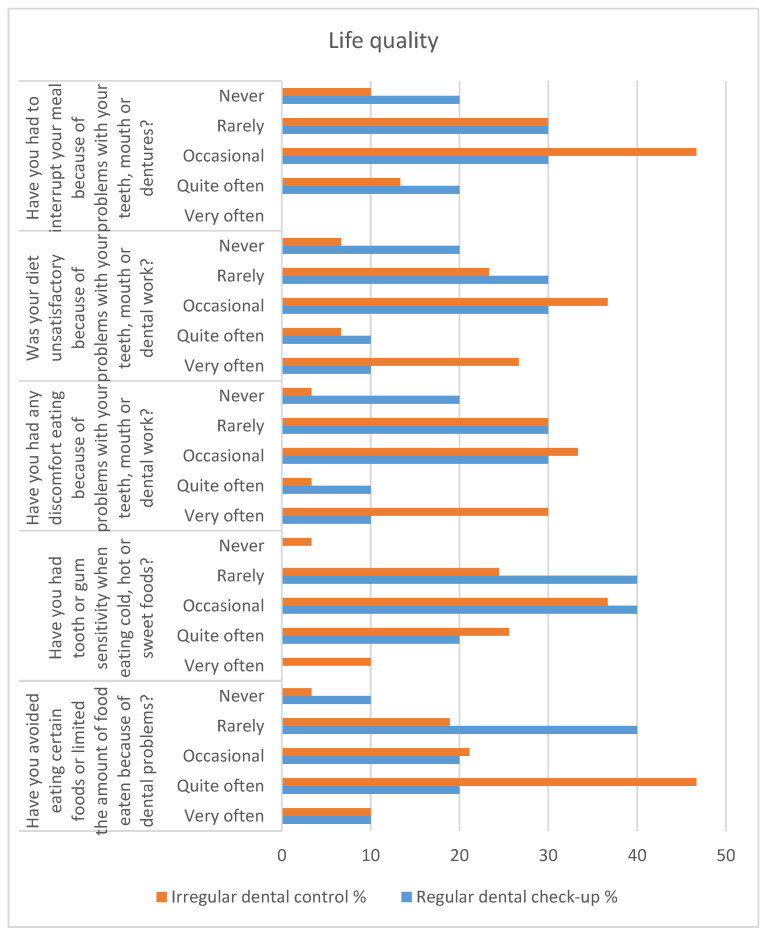
Quality of life from the perspective of dental health in elderly patients, depending on their attendance at periodic dental checkups.

**Figure 2:**
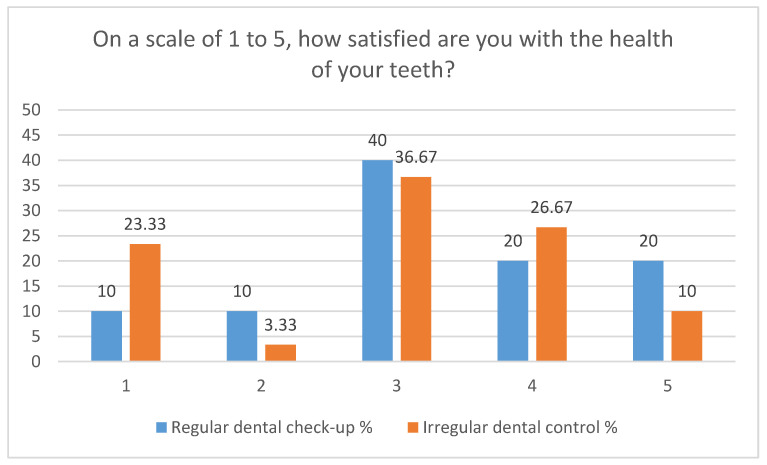
Self-assessment of elderly patients regarding satisfaction with dental health, depending on their attendance at periodic dental checkups.

The patients’ perception of the influence of dental problems on the quality of life was obtained by analyzing the answers to five questions that specifically focused on difficulties in eating food (Figure 1), and a question related to the self-assessment of dental health (Figure 2).

To the question “Have you avoided eating certain foods or limited the amount of food eaten because of dental problems?” 40% of patients who present themselves regularly for periodic checkups claim “very rarely” vs. 18.89% of patients with irregular control. The ratio of answers reverses for the answer “quite often” (20% vs. 46.67%). The occurrence of tooth sensitivity when eating cold, hot, or sweet foods is mentioned “very often” only by patients who present irregularly for dental checkups (10%) (*p* = 0.07). The answer “very rarely” is given by 40% of patients who regularly present themselves for dental checkups vs. 24.44% of patients with irregular dental control, and for the answer “occasionally”, the data are similar (40% vs. 36.67%). Another question: “Have you had any discomfort eating because of problems with your teeth, mouth or dental work?” concerned the influence of dental problems on general health. A statistically significant difference was obtained for the answer “never” where 20% of patients who present themselves for regular checkups do not report concerns vs. 3.33% of patients with irregular controls. The answers “occasionally” and “very rarely” are found in approximately 60% of patients and are similar in the two groups (60% vs. 63.33%). Problems generated by oral pathology led to an unsatisfactory diet “very often” in 10% of patients presenting regularly for control vs. 26.67% of patients with irregular controls and “quite often” at 10%—regular control vs. 6.67%—irregular control. Problems with the teeth, mouth, or dental prosthesis caused the interruption of a meal “quite often” in 20% of patients with regular checkups vs. 13.33%—irregular and “occasional” interruptions in 30% of patients with regular checkups vs. 46.67%—irregular checkups. The self-assessment of satisfaction with dental health, on a scale from 1 (very dissatisfied) to 5 (very satisfied), is satisfactory (scale 3–5) for 80% of patients with regular checkups vs. 73.33% of patients with irregular controls. “Very dissatisfied”—scale 1 is reported by 10% of patients who present themselves regularly for checkups vs. 23.33% of those with irregular checkups. Patients who declare themselves “Very satisfied”—scale 5 are in a percentage of 20% with regular control vs. 10% with irregular control.

### 3.6. Dental and Endodontic Diagnosis

The collaboration with the dentist in carrying out the study outlined the data perceived by the patients with the real dental health problems of the elderly patients admitted to the study. At the endoral examination, no statistically significant differences were obtained between the two groups regarding the odontal diagnosis. Simple caries are present in 20% of patients who present regularly for control vs. 13.33% in patients with irregular checkups, and complicated caries are found in 50% of patients who present regularly for checkups vs. 53.33% in patients with irregular controls. 

Endodontic diagnosis reflects the following conditions in elderly patients, comparing regular control with irregular control: inflammatory hyperemia—statistically significant differences (20% vs. 41.11%, *p* = 0.03), pulpitis (26.67% vs. 34.44%), apical periodontitis (70% vs. 74.44%), pulp necrosis (13.33 vs. 26.67%), and pulp gangrene (30% vs. 37.78%). Statistically significant differences are found in acute apical periodontitis, present in 80.95% of patients with periodic checkups vs. 46.27% patients with irregular controls (*p* = 0.001), and in chronic apical periodontitis, more frequently in patients with irregular controls (19.05% vs. 53.73%, *p* = 0.001) (Tabel 5).

From the point of view of the prosthetic diagnosis, there are differences between the type of dentition of the two categories of patients: in those who regularly go to the dentist, class III dentitions predominate in a percentage of 60% compared to 38.89% in patients who go irregularly (*p* = 0.04). In patients who do not go to the dentist, class I and II edentities are statistically higher, amounting to 50% of the total, compared to 40% of the total of patients with class I and II edentities who present themselves regularly for control. Statistically significant differences were not found in the case of prosthetic works; the patients with regular control who presented prosthetic works were in the percentage of 70% compared to 63.33% in the case of patients from the second batch (Table 5).

Regarding the periodontal diagnosis, differences were recorded in the presence of corneous marginal periodontitis, where regular control vs. irregular control was 40% vs. 60%.

## 4. Discussion

This exploratory cross-sectional study aimed to determine whether elderly patients who regularly present themselves for periodic dental checkups have a better oral health status than those who visit the dentist only when necessary, having irregular dental checkups.

Similar studies published over time in the field show the importance given to the oral health of the elderly population by practitioners, researchers, or decision-making institutions in health [19,20,21,22,23,24,25,26]. Although an important component of oral health policies and health education is prophylaxis, in which the population is recommended a regular dental checkup every 6 months starting from childhood, unfortunately, there are many people who access oral health services only when a problem/emergency occurs or when, due to tooth loss, feeding problems occur. Notwithstanding oral hygiene products, which reinforce the information provided by the specialists, on analyzing the published data on oral pathology, we note that there is no improvement in oral morbidity indicators [11,27,28].

The elderly population is the most exposed to risk factors due to the accumulation of factors acquired during life that lead to the alteration of systemic health status and, implicitly, to the alteration of oral health. Additionally, in many cases, oral pathology is responsible for the appearance of systemic conditions [29,30,31]. The direct relationship between general health and oral health is carefully followed by dentists considering diagnostic and therapeutic conduct as well as the evolution of disease [32,33].

From the study carried out and from the descriptive analysis of the selected study group describing a series of statistically significant differences between people who regularly visit the dentist versus those who present irregularly, only 25% of the patients attended regular dental consultations. Thus, there are differences in terms of age (the more advanced the age, the greater the proportion of those who do not present themselves periodically and regularly for dental checkups), educational level (those with higher education present in a larger proportion for regular periodical checkups) and socioeconomic status through income (patients with low incomes presenting irregularly for dental checkups).

From the point of view of morbidity, by analyzing the chronic diseases present, a large proportion of the elderly patients indicate the existence of some conditions; similar results were obtained in other studies [34,35,36,37]. Dental conditions cause additional medication consumption, which in some cases may lead to drug interactions if medications are administered without a medical prescription [38,39]. Patients who regularly visit the dentist have statistically significantly lower drug consumption for dental pain in the last month compared to those who do not visit the dentist regularly.

The period of use of a toothbrush is recommended as a maximum of 3 months. There is also a series of preventive treatments with considerable results in terms of avoiding the development of dental diseases [40,41,42]. All these basic recommendations are known by most people, but, unfortunately, they are not always followed. Most patients, when completing the questionnaire, affirmatively know these basic rules, but when they are asked to answer the questions, they admit that they do not always follow the recommendations, citing a number of reasons in this regard. Following the study, a better performance of dental hygiene is observed among patients who regularly see the dentist. It is alarming that we find patients (10% among those who do not visit the dentist regularly) who do not use a toothbrush and toothpaste, and approximately 40% of the patients enrolled in the study do not know which toothpaste they use.

Patient accessibility to dental medical services is limited due to low funds allocated to dentistry in the health insurance system [43,44,45]. The dental healthcare services available to patients are those included in the minimum service package (with general access for emergency treatment) and the basic medical service package (available only to insured patients). Another impediment is the fact that only a small number of dentists are in contractual relations with the health insurance company [46]. In this context, patients who present themselves for dental medical checkups are forced to pay this for service directly to the dentist, thus creating a major financial barrier in the access of insured patients to such services. From the results of the analysis carried out, approximately 40% of the interviewed patients indicate a lack of money as the main reason for presenting to the dentist only when necessary. Difficult access is mentioned by 10% of the group of patients who regularly visit the dentist and who come from rural areas.

Attendance is largely conditioned by patients’ perception of their own health and the extent to which the health system can meet their health needs. From the studies carried out, regarding oral medical prevention at the international level, Romanians do not attach importance to oral health; they do not have a culture of medical prevention, and the number of consultations with the dentist, at less than one consultation per year, places Romania in last place in the international ranking [47]. The absence of acute dental symptoms during the pandemic determined the decrease in the number of patients who presented themselves to the dentist. Past unpleasant experiences, a lack of perceived need, and fear are particularly important factors in the frequency of visits to the dental office [48]. An analysis carried out in the Republic of Moldova shows that, in the period 2008–2012, a constant share of 7% of patients benefited from dental consultations, and that, in 2016, only 3.7% of the total population had visited the dentist in the last 4 weeks [49]; in 2021 the share rose to 8% [50].

The importance of periodic dental control is significantly different in the two groups; all patients in the regular group consider it important vs. 80% of the batch with irregular controls.

Oral health is an important component of general health, having a predominant role in the quality of life. Poor oral health has a negative impact on the quality of life through discomfort, pain, and suffering, thus causing functional, aesthetic, nutritional, and psychological problems [51,52].

Dental health influences the quality of life of patients, with differences between the regular control/irregular control groups. Thus, patients who present irregularly for dental checkups encounter a series of problems due to dental pathology: they avoid the consumption of food relatively often (46.67%) and exhibit dental sensitivity to the consumption of cold, hot, or sweet foods, very often in a proportion of 10%. Differences also appear in terms of feeding discomfort, where 20% of patients with regular checkups declare that they have never encountered problems vs. 3.33% of patients with irregular checkups. 

Elderly patients using dental services are more aware of their oral health, which also implies better dental hygiene [53]. Correct and accurate odontal and endodontic diagnosis is essential for successful treatment in medicine and dentistry. In elderly patients, accurate diagnosis is imperative to identify dental health problems. Periodontal disease encompasses a variety of phenotypes defined by signs and symptoms, thus constituting the periodontal syndrome. The evolution is most often progressive and irreversible, the disease being associated with disability, decreased quality of life, and high care costs [54,55]. 

Periodontal disease has an important social character, and can be considered a public health problem. Its appearance and propagation are related to age, sex, occupation, social level, and education. Our study proves that this is true, with 60% of the respondents who visit the dentist when needed suffering from periodontal disease and 50% of them suffering from class I and II dentitions.

The better oral health status of patients who present themselves regularly for periodic checkups confirms the need for health education over treatment to inform and raise awareness, and to empower older people to practice prevention and develop attitudes favorable to accepting appropriate treatment within regular dental and medical checkups.

Limitations of the study. The period for establishing the study group and enrolling patients overlaps with the epidemiological state of alert established in Romania due to the pandemic epidemiological situation caused by the increased incidence of cases of SARS-CoV-2 infection [56]. The vast majority of dentists who provide dental medical services carry out their activity in the private system, so a large number of them did not give their consent to participate in this study, which considerably limited the number of patients participating in the study. Another obstacle in defining the study group was encountered in the patients from whom we did not obtain consent due to a series of reasons, the most frequently cited ones being the lack of time and the need to solve their health problem as quickly as possible and to leave their offices in order not to be exposed to a potential infection with SARS-CoV-2. Thus, the delimitation of the patients into the two study groups having as a basic criterion periodic dental checkups at the dentist was established after completing the questionnaires, which revealed a considerable difference between patients who present themselves regularly for regular periodic dental checkups and those who visit the dentist’s office only when the state of their dental health requires it (i.e., in a dental emergency).

## 5. Conclusions

The results of the study demonstrated that elderly patients who regularly attend periodic dental checkups have better self-reported and dentist-confirmed oral health status. Descriptively, in the group with regular controls, the proportion of patients decreases with increasing age, and patients with higher education and higher financial resources predominate. The general and oral health status of the elderly population is poor, necessitating the administration of drugs for dental pain in patients who do not regularly attend dental checkups. Dental hygiene is deficient in patients who visit the dentist irregularly, with visits to the dentist being affected by a lack of money and difficult access, but also by unpleasant experiences in the past, a lack of perceived need, and fear. Dental problems influence the state of health in general and implicitly the quality of oral life, producing difficulties in mastication, which lead to poor nutrition, with effects on the general quality of the life of patients. Elderly patients with regular checkups are more aware of their oral health, having a greater responsibility for, and expectations of, their own dental health. 

It is imperative to change the contractual clauses and the legislation related to the dental medical services offered in the health insurance system, with supervision of, and improvement in, oral health through multidisciplinary teams, as well as the provision of the means to restore oral functions within regular periodical dental checkups.

## Figures and Tables

**Table 1:** Epidemiological profile of elderly patients, according to age group (years), education, monthly income (EUR), and presentation to regular dental checkups.

Questionnaire. Section 1		Periodic Dental Control						* *p*	Chi^2^ (df)
		Periodic		Non-Periodic		Total			
		No.	%	No.	%	No.	%		
Age (years)	65–69	15	50.00	65	72.22	80	66.67	0.02	18.462 (4)
	70–74	15	50.00	9	10.00	24	20.00	* <0.001	
	75–79	0	0	9	10.00	9	7.50	* 0.07	
	80–84	0	0	6	6.67	6	5.00	0.14	
	85–89	0	0	1	1.11	1	0.83	0.56	
Gender	female	19	63.3	50	55.5	69	57.5	* <0.001	1.327 (10)
	male	11	26.7	40	44.5	51	42.5	* <0.001	
Environment	urban	23	76.7	34	37.8	57	47.5	* <0.001	1.273 (1)
	rural	7	23.3	56	62.2	63	52.5	* <0.001	
Education	primary	0	0	6	6.67	6	5.00	0.14	3.528 (2)
	medium	15	50.00	69	76.67	84	70.00	* 0.006	
	higher	15	50.00	15	16.67	30	25.00	* 0.003	
Income (EUR)	<100	3	10.00	0	0.00	3	2.50	* 0.002	6.672 (3)
	100–300	6	20.00	45	50.00	51	42.50	* 0.004	
	300–600	18	60.00	42	46.67	60	50.00	0.2	
	600–1000	3	10.00	3	3.33	6	5.00	0.14	

* *p* < 0.05 (statistically significant).

**Table 2:** Associated chronic diseases in elderly patients, according to their presentation at regular dental checkups.

Questionnaire. Section 2			Periodic Dental Control						* *p*	Chi^2^ (df)
			Periodic		Non-Periodic		Total			
			No.	%	No.	%	No.	%		
Chronic diseases	Yes		12	40.00	48	53.33	60	50.00	0.2	1.200 (1)
	No		18	60.00	42	46.67	60	50.00		
Type of chronic diseases	respiratory		0	0	3	3.33	3	5.00	0.31	31.250 (5)
	cardiovascular		9	30.00	33	36.67	42	70.00	0.50	
	oncologic		0	0	3	3.33	3	5,00	0.31	
	gastrointestinal		3	10.00	3	3.33	6	10.00	0.14	
	endocrinologic		0	0	3	3.33	3	5.00	0.31	
	rheumatologic		0	0	3	3.33	3	5.00	0.31	
Daily consumption of drugs		Yes	18	60.00	54	60.00	72	60.00	1	1.125 (1)
		No	12	40.00	36	40.00	48	40.00		
Number of drugs administered daily	Between 1 and 3		12	40.00	33	36.67	45	63.61	0.74	8.909 (3)
	Between 4 and 7		6	20.00	12	13.33	18	25.00	0.37	
	Between 7 and 10		0	0	3	3.33	3	4.16	0.31	
	Over 11		0	0	6	6.67	6	8.33	0.14	
Consumption of drugs for dental pain last month		Yes	6	20.00	33	36.67	39	32.50	0.09	1.845 (1)
		No	24	80.00	57	63.33	81	67.50		

* *p* < 0.05 (statistically significant).

**Table 3:** Oral hygiene in elderly patients, depending on their attendance at periodic dental checkups.

Questionnaire. Section 3		Periodic Dental Control						* *p*	Chi^2^ (df)
		Periodic		Non-Periodic		Total			
		No.	%	No.	%	No.	%		
Dental hygiene—products	only with water/rinse the mouth	0	0	9	10.00	9	7.50	0.07	18.087 (3)
	with toothpaste and toothbrush	30	100	72	80.00	102	8.50	* 0.008	
	I use mouthwash	3	10.00	3	3.33	6	5.00	1	
	I use dental floss	0	0	3	3.33	3	2.500	0.31	
	I use toothpicks	0	0	0	0	0	0	1	
Dental hygiene—frequency	daily	30	100	75	83.33	105	87.50	* 0.01	20.957 (3)
	1 time a day	18	60.00	66	73.33	84	70.00	0.16	
	2 times a day	12	40.00	9	10.00	21	17.50	* 0.000	
	after every meal	0	0	0	0	0	0	1	
	occasionally	0	0	15	16.67	15	12.50	* 0.01	
Brushing your teeth in the evening	Never	0	0	0	0	0	0	1	14.609 (3)
	Very rare	6	20.00	30	33.33	36	30.00	* 0.017	
	Once in a while	3	10.00	27	30.00	30	25.00	* 0.02	
	Almost every night	6	20.00	15	16.67	21	17.50	0.67	
	Every night	15	50.00	18	20.00	33	27.50	* 0.001	
The toothbrush used is	Hard/rough	3	10.00	3	3.33	6	5.00	0.14	9.870 (4)
	Medium	18	60.00	51	56.67	69	57.50	0.75	
	Soft	6	20.00	6	6.67	12	10.00	* 0.03	
	Electric	0	0	0	0	0	0	1	
	I do not know	3	10.00	21	23.33	24	20.00	0.11	
	Non-respondents	0	0	9	10.00	9	7.50	0.07	
How often do you change your toothbrush?	At 3 months	15	50.00	24	26.67	39	32.50	* 0.01	9.636 (3)
	Once a year	0	0	12	13.33	12	10.00	* 0.03	
	When needed	15	50.00	45	50.00	60	50.00	1	
	Non-respondents	0	0	9	10.00	9	7.50	0.07	
Used toothpaste	With fluoride	9	30.00	18	20.00	27	22.50	0.25	16.909 (4)
	Without fluoride	0	0	3	3.33	3	2.50	0.31	
	Special for gums	9	30.00	21	23.33	30	25.00	0.46	
	I do not know	12	40.00	39	43.33	41	34.16	0.75	
	Non-respondents	0	0	9	10.00	9	7.50	0.07	
Bleeding occurs when brushing teeth	Yes	12	40.00	42	46.67	54	45.00	0.52 0.54	8.364 (2)
	No	18	60.00	39	43.33	57	47.50		
	Non-respondents	0	0	9	10.00	9	7.50	0.07	
Did you have dental problems as a child?	Never	0	0	3	3.33	3	2.50	0.31	14.043 (4)
	Rarely	3	10.00	24	26.67	27	22.50	* 0.05	
	Sometimes	15	50.00	27	30.00	42	35.00	* 0.04	
	Often	9	30.00	33	36.67	42	35.00	0.5	
	Very often	3	10.00	3	3.33	6	5.00	0.14	
Where you can find out what you need to know about oral health	From the dentist	24	80.00	48	53.33	72	60.00	* 0.01	17.783 (4)
	From the TV	6	20.00	24	26.67	30	25.00	0.46	
	From the internet	0	0	6	6.67	6	5.00	0.14	
	Other sources	0	0	6	6.67	6	5.00	0.14	
	Non-respondents	0	0	6	6.67	6	5.00	0.14	

* *p* < 0.05 (statistically significant).

**Table 4:** Elderly patients’ accessibility to, and attendance at, the dentist, depending on their presentation for regular dental checkups.

Questionnaire			Periodic Dental Control						* *p*	Chi^2^ (df)
			Periodic		Non-Periodic		Total			
			No.	%	No.	%	No.	%		
What prevents you from getting to the dentist as often as you would like?	Fear		3	10.00	21	23.33	24	20.00	0.11	12.45 (5)
	Unpleasant experiences in the past		6	20.00	9	10.00	15	12.50	0.15	
	Difficult access		0	0	9	10.00	9	7.50	0.07	
	Lack of money		12	40.00	21	23.33	24	20.00	0.07	
	It is not necessary		9	30.00	24	26.67	33	27.50	0.72	
	Non-respondents		0	0	6	6.67	6	5.00	0.14	
Why do you go to the dentist more often?	Toothache		9	30.00	42	46.67	51	4,50	0.11	23.618 (8)
	Gum pain		3	10.00	6	6.67	9	7.50	0.55	
	Bleeding gums		3	10.00	0	0	3	2.50	* 0.002	
	Scaling		0	0	0	0	0	0	1	
	Caries treatment		6	20.00	9	10.00	15	12.50	0.15	
	Dental extractions		3	10.00	6	6.67	12	10.00	0.55	
	Prosthetic works		6	20.00	6	6.67	12	10.00	* 0.03	
	Problems with dentures		0	0	15	16.67	15	12.50	* 0.01	
	Non-respondents		0	0	6	6.67	6	5.00	0.14	
Do you usually go to the same dentist?		Yes	27	90.00	75	83.33	103	85.83	0.37	6.358 (2)
		No	3	10.00	9	10.00	12	10.00	1	
		Non-respondents	0	0	6	6.67	6	5.00	0.14	
Do you consider regular checkups at the dentist important?		Yes	30	100	72	80.00	102	85.00	* 0.008	5.289 (2)
		No	0	0	9	10.00	9	7.50	0.07	
		Non-respondents	0	0	9	10.00	9	7.50	0.07	

* *p* < 0.05 (statistically significant).

**Table 5:** Dental and endodontic diagnosis in elderly patients, depending on their presentation at regular dental checkups.

Questionnaire			Periodic Dental Control						* *p*	Chi^2^ (df)
			Periodic		Non-Periodic		Total			
			Nr.	%	Nr.	%	No.	%		
Dental diagnosis	Simple caries	Yes	6	20	12	13.33	18	15.00	0.37	1.537 (1)
		No	24	80	78	83.33	102	85.00		
	Complicated caries	Yes	15	50	48	53.33	63	52.50	0.75	1.397 (1)
		No	15	50	39	43.33	54	45.00		
Endodontic diagnosis	Inflammatory hyperemia	Yes	6	20	37	41.11	43	35.83	* 0.03	1.386 (1)
		No	24	80	53	58.89	77	64.17		
	Pulpitis	Yes	8	26.67	31	34.44	39	32.50	0.43	6.341 (3)
		No	22	73.33	59	65.56	81	67.50		
		Serous	5	62.5	17	54.83	22	18.33	0.46	
		Purulent	3	37.5	14	45.17	17	14.17	0.46	
	Apical periodontitis	Yes	21	70	67	74.44	88	73.33	0.63	5.498 (3)
		No	9	30	23	25.56	32	26.67		
		Acute	17	80.95	31	46.27	48	40.00	* 0.001	
		Chronic	4	19.05	36	53.73	40	33.33	* 0.001	
	Pulp necrosis	Yes	4	13.33	24	26.67	28	23.33	0.13	1.382 (1)
		No	26	86.67	66	73.33	92	76.67		
	Pulpal gangrene	Yes	9	30	34	37.78	43	35.83	0.44	1.417 (1)
		No	21	70	56	62.22	77	64.16		
Prosthetic diagnosis	The edentation class	1	3	10.00	27	30.00	30	25.00	* 0.02	4.387 (3)
		2	9	30.00	18	20.00	27	22.50	0.25	
		3	18	60.00	42	46.67	60	50.00	* 0.04	
		6	0	0.00	3	3.33	3	2.50	0.33	
	Prosthetic works	Yes	21	70.00	57	63.33	78	65.00	0.5	1.265 (1)
		No	9	30.00	33	36.67	42	35.00		
Periodontal diagnosis	Simple chronic gingivitis	Yes	9	30.00	30	33.33	39	32.50	0.73	1.421 (1)
		No	21	70.00	60	66.67	81	67.50		
	Chronic marginal periodontitis	Yes	12	40.00	54	60.00	66	55.00	0.05	1.257 (1)
		No	18	60.00	36	40.00	54	45.00		

* *p* < 0.05 (statistically significant).
